# Quiescent Fibroblasts Are More Active in Mounting Robust Inflammatory Responses Than Proliferative Fibroblasts

**DOI:** 10.1371/journal.pone.0049232

**Published:** 2012-11-14

**Authors:** Bo-Rui Chen, Huei-Hsuan Cheng, Wei-Chung Lin, Kai-Hsuan Wang, Jun-Yang Liou, Pei-Feng Chen, Kenneth K. Wu

**Affiliations:** 1 Institute of Cellular and System Medicine, National Health Research Institutes, Zhunan, Miaoli, Taiwan; 2 Institute of Biotechnology, National Tsing Hua University, Hsin-Chu, Taiwan; Kaohsiung Chang Gung Memorial Hospital, Taiwan

## Abstract

Quiescent cells are considered to be dormant. However, recent studies suggest that quiescent fibroblasts possess active metabolic profile and certain functional characteristics. We previously observed that serum-starved quiescent fibroblasts respond to proinflammatory stimuli by robust expression of cyclooxygenase-2 (COX-2), which declines after the quiescent fibroblasts are driven to proliferation. In this study, we elucidated the underlying signaling and transcriptional mechanism and identified by microarray genes with similar differential expression. By using pharmacological inhibitors coupled with gene silencing, we uncovered the key role of protein kinase C δ (PKCδ) and extracellular signal regulated protein kinase 1/2 (ERK1/2) signaling in mediating COX-2 expression in quiescent cells. Surprisingly, COX-2 expression in proliferative cells was not blocked by PKCδ or ERK1/2 inhibitors due to intrinsic inhibition of PKCδ and ERK1/2 in proliferative cells. Restrained COX-2 transcription in proliferative cells was attributable to reduced NF-κB binding. Microarray analysis identified 35 genes whose expressions were more robust in quiescent than in proliferative cells. A majority of those genes belong to proinflammatory cytokines, chemokines, adhesive molecules and metalloproteinases, which require NF-κB for transcription. Quiescent fibroblasts had a higher migratory activity than proliferative fibroblasts as determined by the transwell assay. Selective COX-2 inhibition reduced migration which was restored by prostaglandin E_2_. As COX-2 and inflammatory mediators induce DNA oxidation, we measured 8-hydroxydeoxyguanosine (8-OHdG) in quiescent vs. proliferative fibroblasts. PMA-induced 8-OHdG accumulation was significantly higher in quiescent than in proliferative fibroblasts. These findings indicate that quiescent fibroblasts (and probably other quiescent cells) are at the forefront in mounting inflammatory responses through expression of an array of proinflammatory genes via the PKCδ/ERK1/2 signaling pathway.

## Introduction

Quiescent cells are traditionally considered to be small, dense cells passively exiting from the cell cycle. Studies on model organisms and mammalian cells have provided evidence for reduced DNA and protein synthesis and low metabolic rates in quiescent cells [1], [2]. Thus, quiescent cells are thought to be dormant waiting for the cues to enter cell cycle to contribute to cell replication. However, several recent reports have challenged this viewpoint. Genetic analysis has shown that quiescent cells are dynamic in gene expression and maintains a unique quiescence program [3]. Furthermore, quiescent fibroblasts exhibit high metabolic rates tilting towards pentose phosphate pathway and NADPH generation [4]. These results suggest that quiescent cells are not dormant and may in fact possess distinct cellular functions. We previously observed that quiescent fibroblasts are highly responsive to stimulation by proinflammatory mediators. In response to stimulation by cytokines, lipopolysaccharides, mitogenic and growth factors, they express a much higher level of a prototypic inflammatory gene, cyclooxygenase-2 (COX-2) [5], [6], than proliferative fibroblasts [7]. However, it is unclear whether other proinflammatory genes are controlled in quiescent vs. proliferative cells in a manner similar to COX-2 nor is it known how the differential response is signaled. In this study, we analyzed the expression profiling by microarray and the signaling pathway via which the differential responses in quiescent vs. proliferative cells are controlled. We have identified cytokines, chemokines, matrix metalloproteinases and adhesive molecules whose expressions are more robust in quiescent than in proliferative cells. Robust COX-2 expression in quiescent fibroblasts in response to stimulation with tumor necrosis factor α (TNFα) or phorbol 12-myristate 13-acetate (PMA) is signaled via protein kinase C δ (PKCδ) and its downstream signaling molecule, extracellular signal regulated protein kinase 1/2 (ERK1/2). A low COX-2 expression in proliferative fibroblasts is attributed to shutdown of the PKCδ→ERK1/2 signaling pathway.

## Results

### COX-2 expression is more robust in quiescent than in proliferative fibroblasts

**Figure 1:**
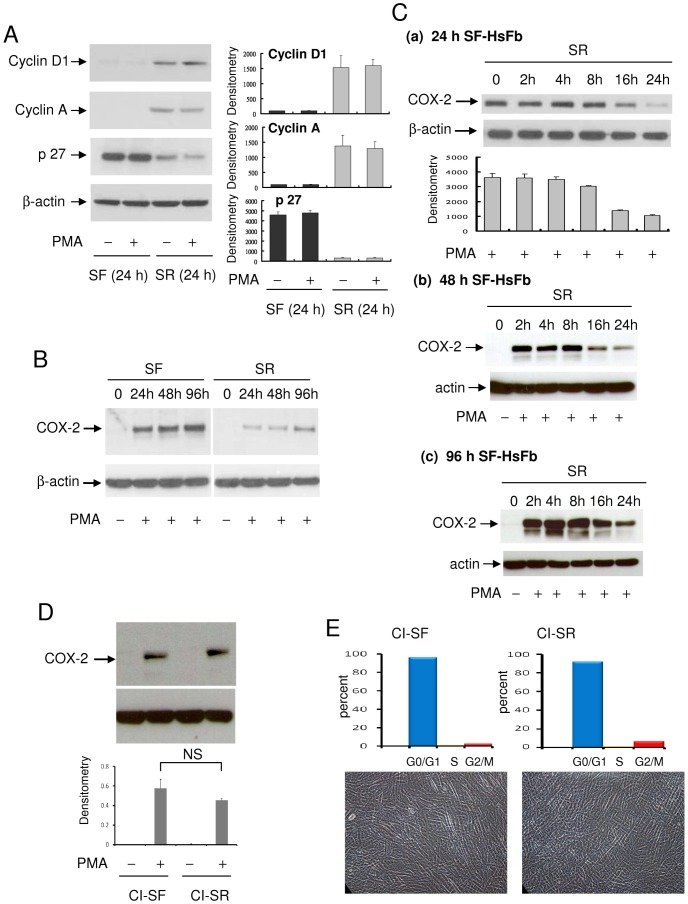
Cell cycle-dependent control of COX-2 expression. (**A**) Cyclin D1, cyclin A and p27 in HsFb cultured in serum-free (SF) medium for 24 h vs. 24 h SR-HsFb were analyzed by Western blotting. SR denotes culture of washed SF-HsFb in medium containing 2.5% fetal bovine serum (FBS). The left panel shows representative blots and the right shows densitometry analysis. The error bars denote mean ± SD (n = 3). (**B**) PMA-induced COX-2 proteins in HsFb cultured in SF medium for 24, 48 or 96 h. The 24 h, 48 h, or 96 h SF-HsFb were washed and incubated in medium containing 2.5% FBS for 24 h. At the indicated time points, PMA (100 nM) was added for 4 h and COX-2 proteins were analyzed by Western blotting. (**C**) Time course of PMA-induced COX-2 expression in (a) 24 h, (b) 48 h and (c) 96 h SF-HsFb replenished with 2.5% FBS for various time periods. At the indicated time point, cells were treated with PMA for 4 h and COX-2 proteins were analyzed by Western blotting. Error bars denote mean ± SEM (n = 3). (**D**) Confluent HsFb cultured in 0.1% FBS for 72 h followed by SF-medium for 24 h (designated contact inhibited SF-HsFb, CI-SF) were washed and replenished with 2.5% FBS for 24 h (designated CI-SR HsFb). CI-SF and CI-SR HsFbs were treated with PMA for 4 h and COX-2 proteins were analyzed. Upper panel shows a representative Western blot and the lower panel mean ± SEM of densitometry of Western blots (n = 3). (**E**) Cell cycle analysis by flow cytometry. Upper panel, distribution of G0/G, S and Gs/M cells and lower panel, photograph of CI-SF vs. CI-SR HsFb. Nagnification: ×200.

Human foreskin fibroblasts (HsFb) were previously established as a cell model for studying cell cycle-dependent cellular and genetic changes [3], [4], [8]. Cell cycle analysis by flow cytometry revealed that a vast majority of HsFb (>90%) cultured in serum-free (SF) medium for 24 h [7] and up to 96 h are at G0/G1 (data not shown). HsFb were driven into proliferation by washing SF-HsFb and incubating them with fresh medium containing 2.5% FBS. Cell cycle of the serum-replenished HsFb (SR-HsFb) was analyzed at regular intervals for 24 h. Cell cycle was unchanged until at 16 h after serum treatment when G0/G1 was reduced to 78% and S-phase was increased to 15%. By 24 h after serum replenishing, G0/G1 was decreased to 22% and S-phase increased to 60% [7]. Cyclin A and cyclin D1, undetectable in the 24 h SF-HsFb, were detected in the 24 h SR-HsFb (Fig. 1A). By contrast, abundant p27 protein was detected in the 24 h SF-HsFb which became markedly declined at 24 h after serum replenishing (Fig. 1A). PMA did not alter the cell cycle-related proteins in SF- or SR-HsFb. PMA-induced COX-2 protein in the 24 h SF-HsFb was >2-fold higher than that in the 24 h SR-HsFb (Fig. 1B). Furthermore, PMA-induced COX-2 expression in the 48 h and 96 h SF-HsFb was as robust as that in the 24 h SF-HsFb which declined to a similar extent after 24 h serum re-stimulation (Fig. 1B).

To analyze the kinetics of decline of PMA-induced COX-2 expression following serum-driven cell cycle progression, 24 h–96 h SF-HsFbs were incubated in medium containing 2.5% FBS and at the indicated time point were treated with PMA for 4 h and COX-2 in the cell lysate was analyzed by Western blotting. Compared to PMA-induced COX-2 expression in 24 h, 48 h or 96 h SF-HsFb, COX-2 level was not reduced until 16 h after serum replenishing when it dropped significantly which was further reduced at 24 h (Fig. 1C). To circumvent the multiple actions of serum, we analyzed PMA-induced COX-2 proteins in serum-starved contact-inhibited HsFb. Cells were seeded at high densities and after they had reached confluency, they were cultured in medium containing 0.1% FBS for 72 h followed by serum-free medium for an additional 24 h. Cells were washed and reincubated in medium containing 2.5% FBS for 24 h. PMA-induced COX-2 protein expression was not significantly reduced in SR-HsFb compared to that in contact inhibited SF-HsFb (Fig. 1D). These results suggest that the contact inhibited SF-HsFb may have entered into “deep” quiescence [3] which is not driven into S-phase by 2.5% FBS. To confirm this, we analyzed cell cycle in contact inhibited SF-HsFb vs. SR-HsFb. Over 90% of cells remained at G0/G1 in SR-HsFb (Fig. 1E). Taken together, these results indicate that the robust PMA-induced COX-2 in quiescent or non-dividing HsFb is suppressed when they enter proliferation.

**Figure 2:**
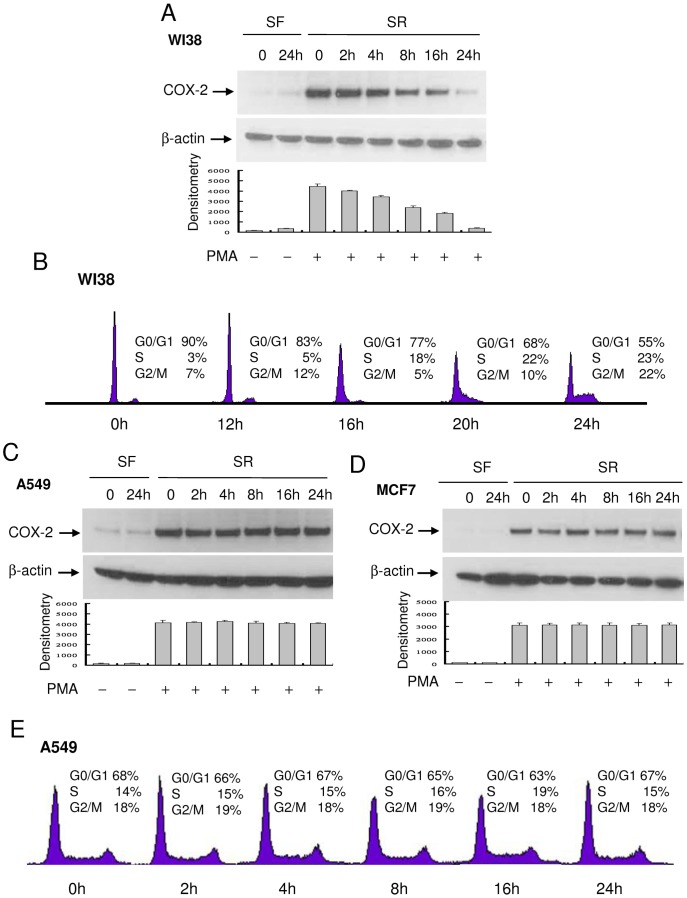
Kinetics of PMA-induced COX-2 expression and cell cycle progression. (**A**) WI38, (**C**) A549 and (**D**) MF7 cells cultured in SF-medium for 24 h were washed and incubated in medium containing 2.5% FBS. At the indicated time point, cells were treated with PMA for 4 h and COX-2 proteins in the cell lysate were analyzed by Western blotting. Upper panels show representative blots and lower panels, densitometry analysis. Error bars indicate mean ± SEM (n = 3). (**B**) and (**E**) WI38 or A549 cells were treated identically as above. At the indicated time points, cells at different phases of cell cycle were analyzed by flow cytometry as previously described [7].

**Figure 3:**
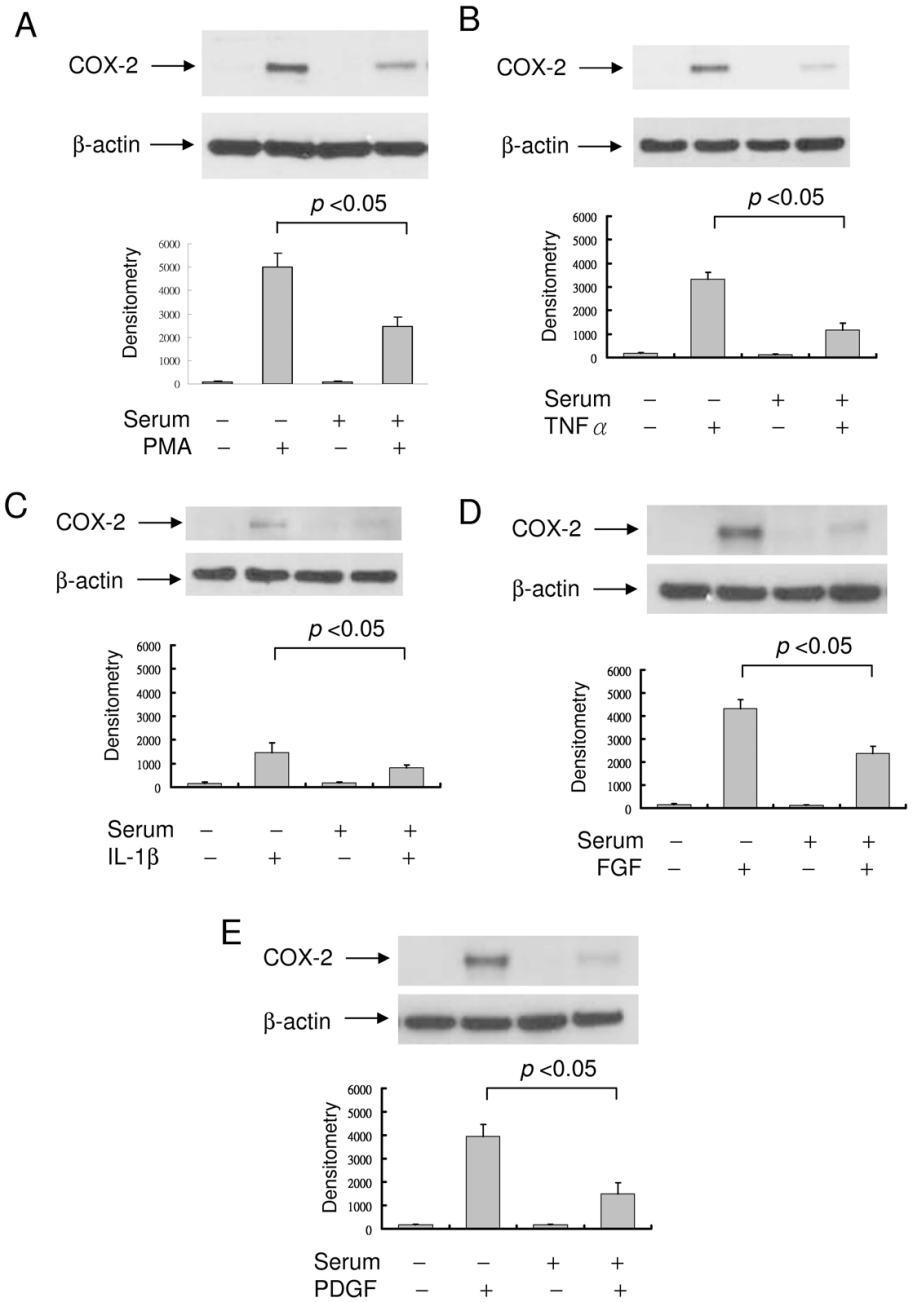
COX-2 expression in SF- vs. SR-HsFb. (**A**) PMA-induced COX-2 proteins in SF- vs. SR-HsFb. Upper panel, representative blot and lower panel, densitometry analysis. Error bars denote mean ± SEM (n = 3). (**B**)**–**(**E**) COX-2 proteins in SF- vs. SR-HsFb treated with TNFα, IL-1β (10 ng/ml), fibroblast growth factor-2 (FGF, 10 ng/ml) or platelet-derived growth factor BB (PDGF, 10 ng/ml) for 4 h. Upper panels show representative Western blots and lower panels, densitometry analysis. Error bars indicate mean ± SEM (n = 3).

The kinetics of PMA-induced COX-2 protein expression in WI38, a lung fibroblast, re-stimulated with FBS was similar to that of HsFb except that COX-2 proteins declined at an earlier time point (8 h) in WI38 than in HsFb (16 h) (Fig. 2A). Cell cycle analysis reveals that WI38 cells entered S-phase at an earlier time point than HsFb (Fig. 2B). By contrast, PMA-induced COX-2 in cancer cells such as A549 cells or MCF7 cells was unchanged by serum re-addition (Fig. 2C and 2D). Cell cycle analysis of A549 cells reveals that a third of the 24 h SF-cells remained at S- and G2/M and percentages of cells in S- and G2/M-phase were not altered by serum re-addition (Fig. 2E). In view of a distinct difference in PMA-induced COX-2 expression in the 24 h SF-HsFb vs. the 24 h SR-HsFb, we performed subsequent experiments in 24 h SF- vs. 24 h SR-cells (designated SF and SR, respectively). TNFα- and IL-1β-induced COX-2 expression declined in SR-HsFb compared to SF-HsFb in a manner similar to PMA-induced COX-2 expression (Fig. 3A–C), so was PDGF- and FGF-induced COX-2 expression (Fig. 3D and 3E).

**Figure 4:**
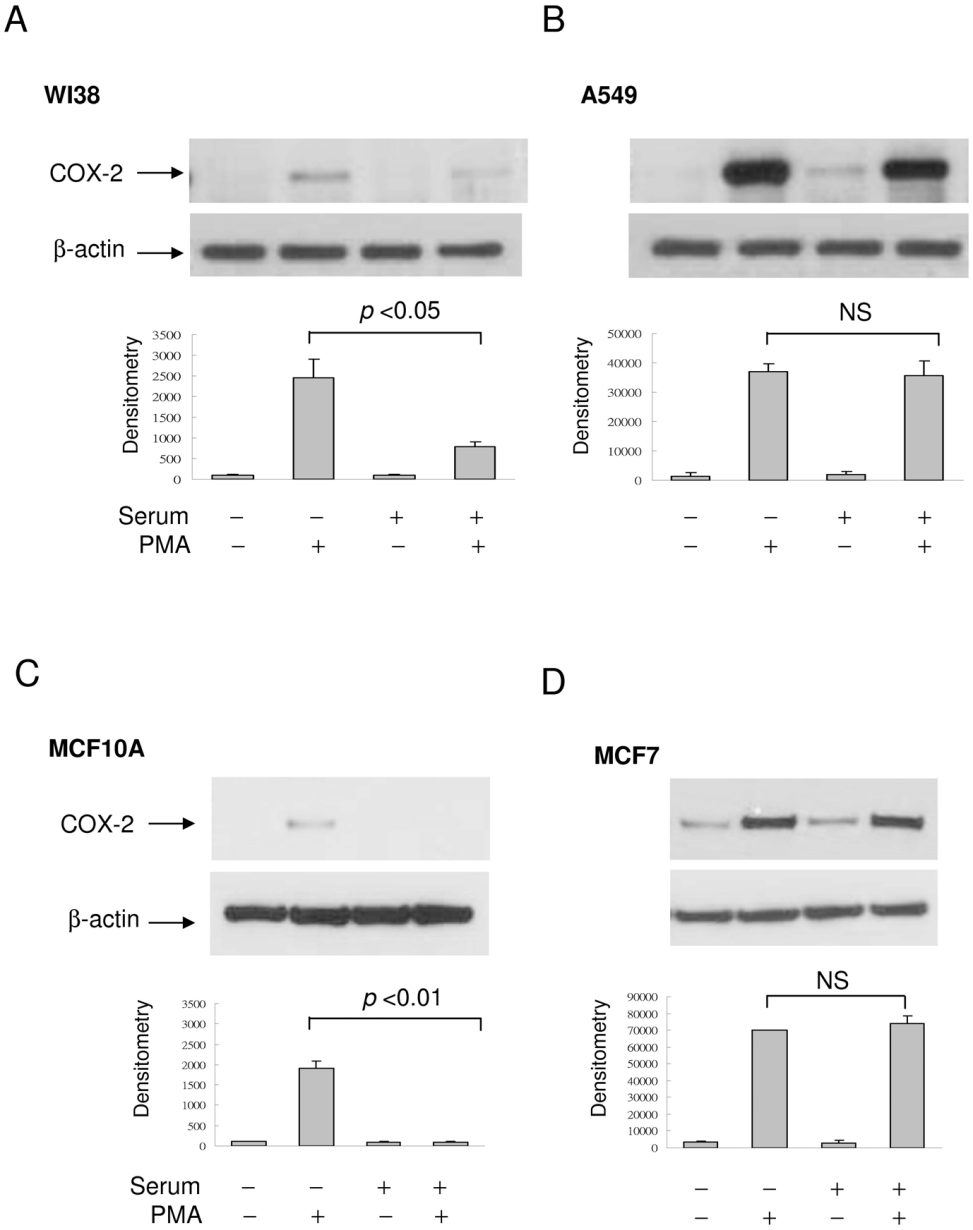
COX-2 expression in cancer vs. normal cells. (**A**)**&**(**B**) PMA-induced COX-2 proteins in SF- & SR-WI38 lung fibroblasts vs. A549 lung cancer cells. (**C**)**&**(**D**) PMA-induced COX-2 expression in SF- & SR-MCF10A breast epithelial cells vs. MCF7 breast cancer cells. The error bars denote mean ± SEM (n = 3).

PMA-induced COX-2 expression in SF-WI38 was >2-fold higher than that in SR-WI38 (Fig. 4A) while the robust PMA-induced COX-2 expression in A549 lung cancer cell was not altered by serum addition (Fig. 4B). To determine whether COX-2 expression was also reduced in proliferative epithelial cells, we compared PMA-induced COX-2 expression in MCF10A, a breast epithelial cell with that in MCF7 breast cancer cell. PMA induced a weak COX-2 band in SF-MCF10A which became undetectable after serum re-addition (Fig. 4C). By contrast, PMA-induced COX-2 in MCF7 was unaffected by serum replenishing (Fig. 4D). These results suggest that in normal cell culture, PMA and cytokine-induced COX-2 expression is more abundant in SF- than in SR-cells while the robust COX-2 expression in cancer cells is not influenced by serum withdrawal or replenishing.

### Higher COX-2 expression in quiescent fibroblasts is attributed to increased COX-2 promoter activity and p65/p50 NF-κB binding

**Figure 5:**
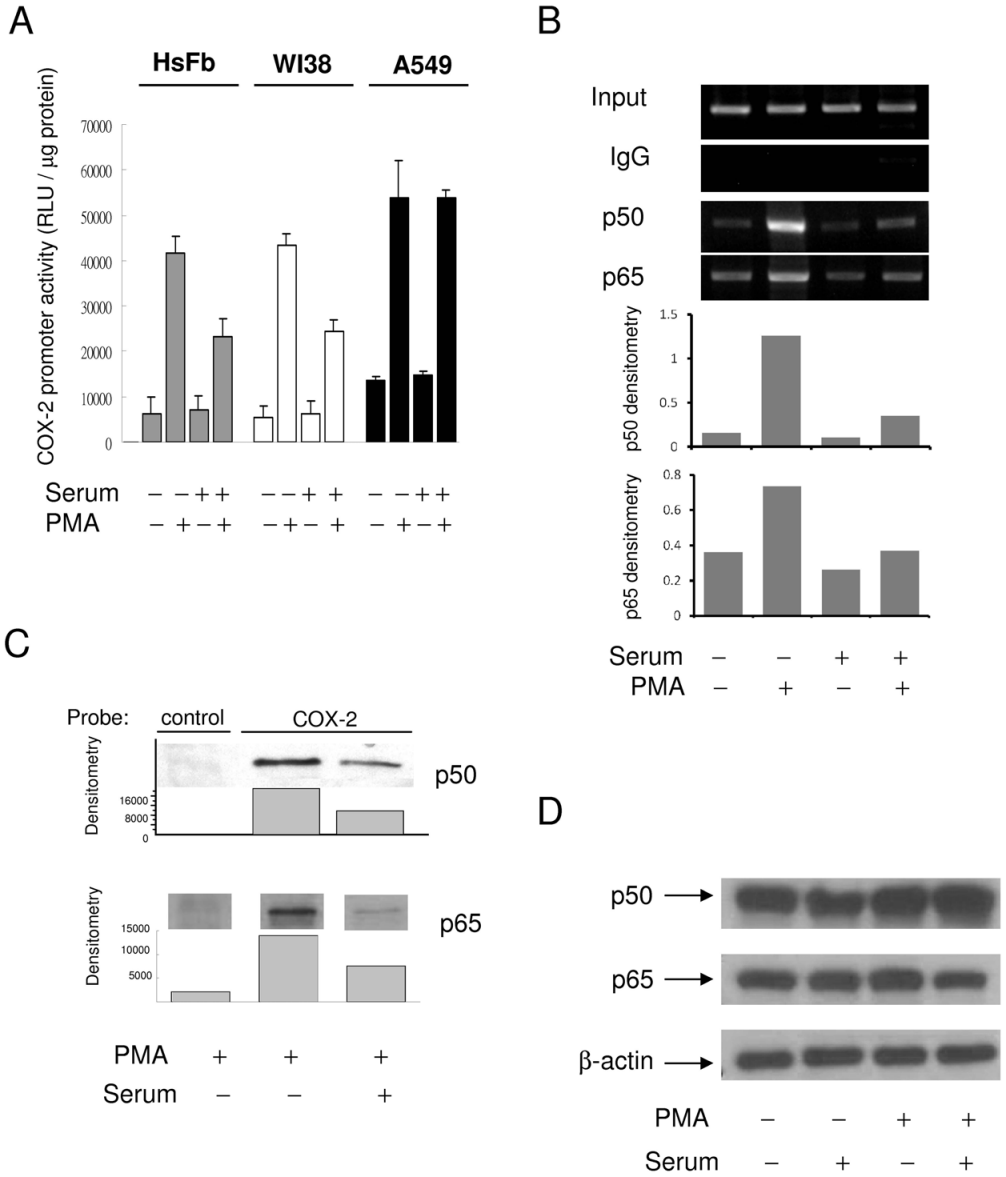
Differential COX-2 transcriptional activation in SF- vs. SR-cells. (**A**) COX-2 promoter activity in SF vs. SR HsFb, WI38 and A549 cells. Each bar denotes mean ± SEM (n = 3). (**B**) and (**C**) NF-κB (p50/p65) binding to COX-2 promoter region analyzed by (**B**) ChIP and (**C**) streptavidin pulldown assay. (**D**) p50 and p65 NF-κB protein levels in SF- vs. SR-HsFb treated with and without PMA.

PMA-induced COX-2 promoter activity in SF-HsFb or SF-WI38 was higher than that in SR-HsFb or SR-WI38 (Fig. 5A). By contrast, PMA-induced COX-2 promoter activity was not different between SR- and SF-A549 cells (Fig. 5A). Since PMA- and proinflammatory cytokines-induced COX-2 expression depends on NF-κB (p65/p50) activation and binding to COX-2 promoter [9], we determined p65/p50 binding by chromatin immunoprecipitation. Indeed, p65 and p50 binding to COX-2 promoter was higher in SF-HsFb than in SR-HsFb (Fig. 5B). PMA-induced p50 and p65 binding to a COX-2 promoter probe was similarly higher in SF-HsFb than in SR-HsFb as analyzed by the streptavidin pulldown assay (Fig. 5C) while the p50 and p65 protein levels were not different between SF- and SR-HsFb (Fig. 5D).

### Differential COX-2 expression in quiescent vs. proliferative fibroblasts is controlled via PKCδ

**Figure 6:**
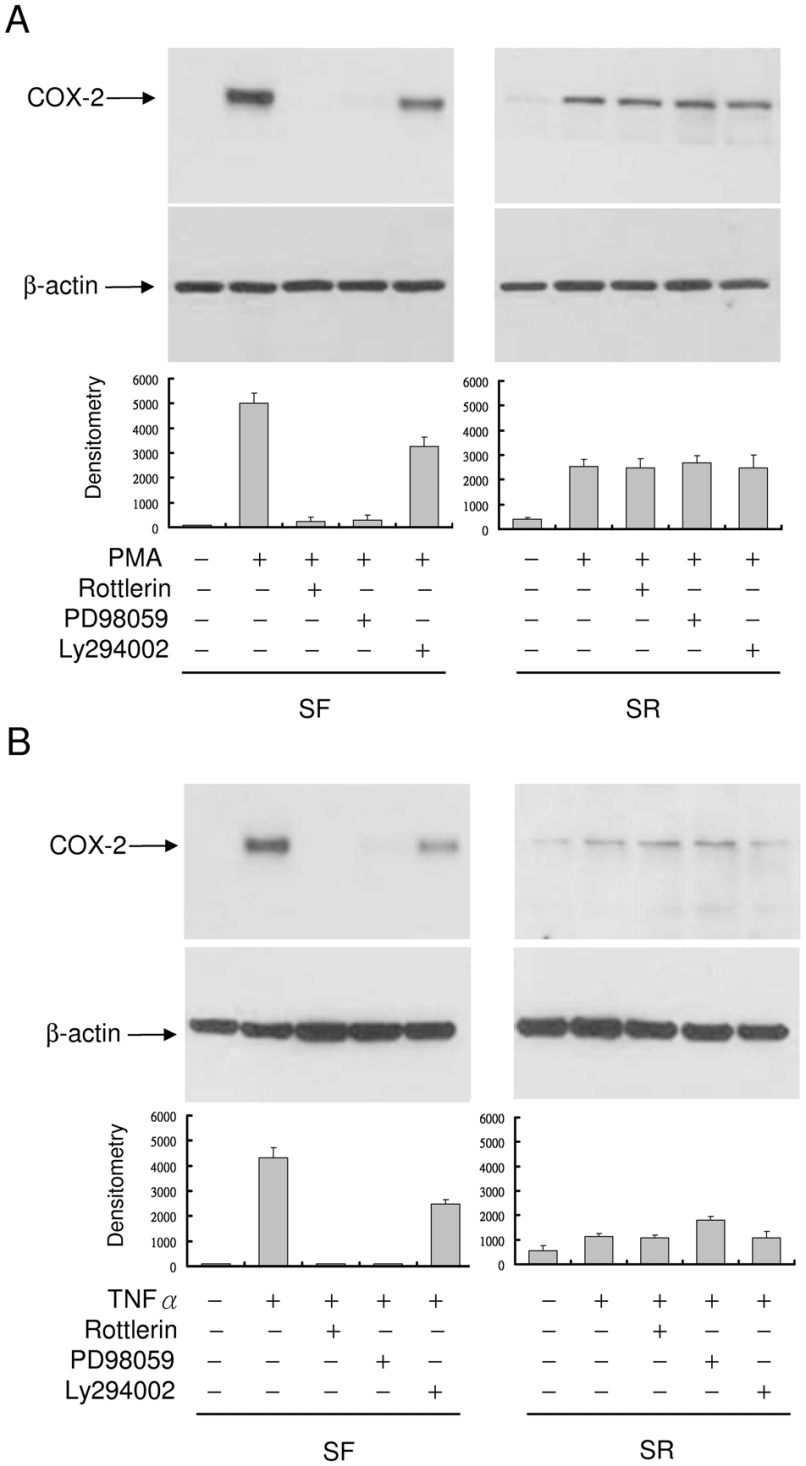
Inhibition of COX-2 expression by rottlerin in SF- but not SR-HsFb. (**A**) SF- or SR-HsFb were pretreated with rottlerin, PD98059 or Ly294002 followed by treatment with PMA for 4 h. COX-2 proteins were analyzed by Western blotting. (**B**) SF- and SR-HsFb were treated with the pharmacological inhibitors followed by TNFα. Upper panels show representative Western blots and the lower panel, densitometry analysis (mean ± SEM, n = 3).

COX-2 transcriptional activation by PMA and proinflammatory cytokines is mediated via several signaling pathways notably PKC and ERK. We suspected that the more robust COX-2 expression in quiescent vs. proliferative fibroblasts might be due to differential activation of the signaling molecules. To test this, we pretreated SF- or SR-HsFb with pharmacological inhibitors of PKC or MEK/ERK1/2 followed by stimulation with PMA or TNFα. PMA-induced COX-2 in SF-HsFb was blocked by rottlerin, a PKCδ inhibitor, or PD98059 (PD), a MEK/ERK inhibitor and only partially affected by Ly294002, a PI-3K inhibitor (Fig. 6A). By contrast, it was unaffected by any of the inhibitors in SR-HsFb (Fig. 6A). TNFα-induced COX-2 expression was similarly inhibited by rottlerin and PD98059 in SF-HsFb but not in SR-HsFb (Fig. 6B). These results suggest that induced COX-2 expression depends on PKCδ and ERK1/2 in quiescent but not proliferative cells.

**Figure 7:**
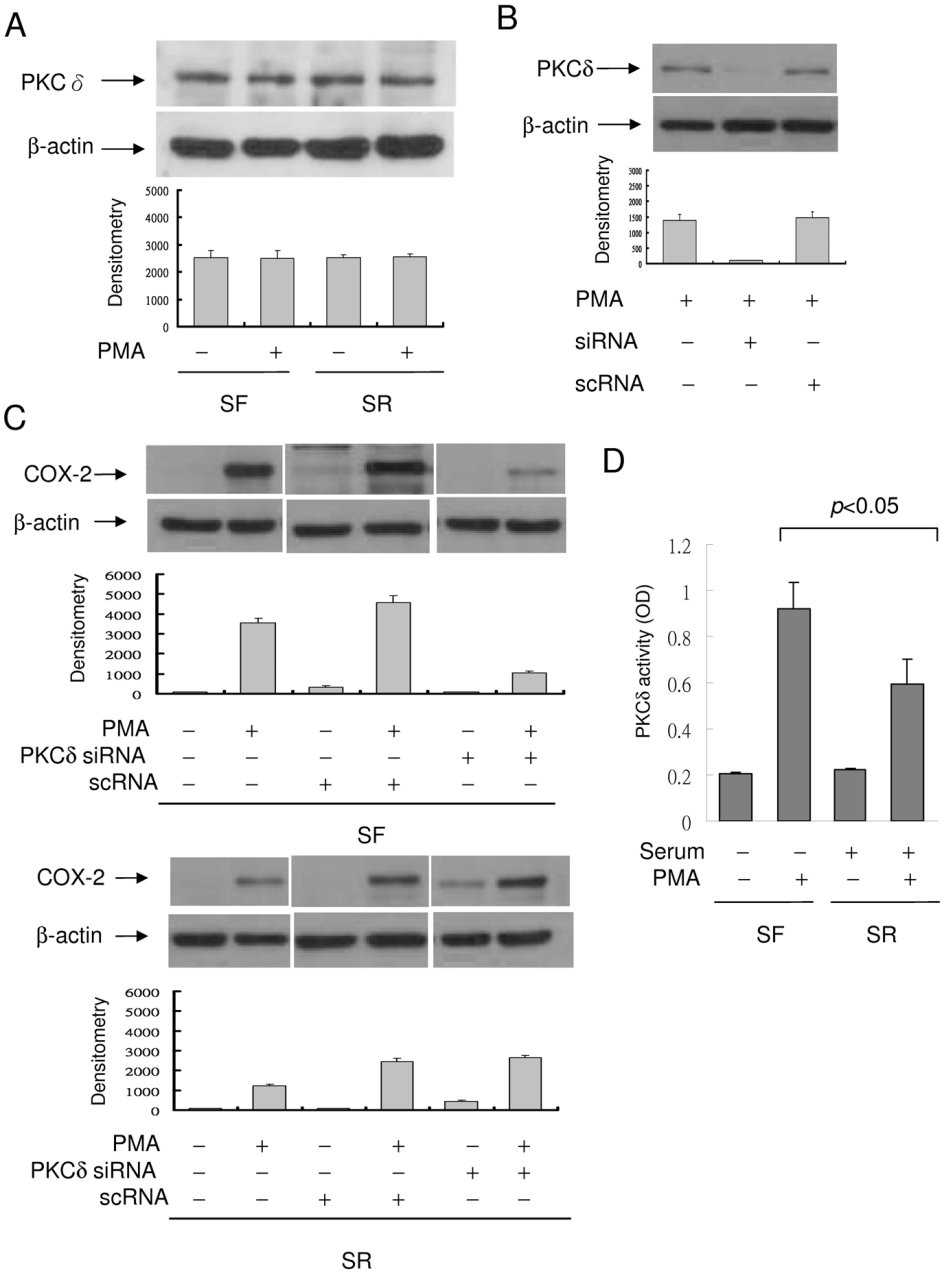
Suppression of PMA-induced COX-2 expression by PKCδ siRNA. (**A**) Analysis of PKCδ protein levels in SF- and SR-HsFb with or without PMA treatment. PKCδ protein level was not different between SF- and SR-cells. (**B**) SF-HsFb were transfected with PKCδ siRNA or a control scRNA. PKCδ expression in the transfected cells was analyzed by Western blotting. (**C**) SF-HsFb (upper panel) and SR-HsFb (lower panel) were transfected with PKCδ siRNA or scRNA. PMA-induced COX-2 expression was analyzed by Western blotting. The blots were quantified by densitometry (mean ± SEM, n = 3). (**D**) PKCδ activity in SF- vs. SR-HsFb treated with PMA was determined by immunoprecipitation (IP) to isolate PKCδ proteins and analyzed the PKC catalytic activity of the IP-isolated PKCδ.

To provide direct evidence for differential involvement of PKCδ in COX-2 expression in quiescent vs. proliferative fibroblasts, we evaluated the effect of PKCδ siRNA on COX-2 expression. PKCδ protein was detected in resting SF- and SR-HsFb at a comparable level which was not increased by PMA (Fig. 7A). PKCδ siRNA completely inhibited PKCδ proteins in PMA-treated fibroblasts while a scrambled control RNA (scRNA) did not (Fig. 7B). Silencing of PKCδ with siRNA resulted in reduction of PMA-induced COX-2 in SF-HsFb (Fig. 7C, upper panel) but not in SR-HsFb (Fig. 7C, lower panel). One possible reason for lack of response of COX-2 expression to PKCδ inhibitor or siRNA is that PKCδ activity is already suppressed in proliferative cells. To evaluate this possible mechanism, we compared PMA-induced PKCδ activation in SF- vs. SR-HsFb. PKCδ was isolated by immunoprecipitation and PKCδ activity of the purified PKCδ was analyzed. The basal PKCδ activity In SF- and SR-HsFb was similar. However, PMA induced a larger increase in PKCδ activity in SF- than in SR-HsFb (Fig. 7D). Thus, it is possible that a low COX-2 expression in proliferative fibroblasts is due to suppression of the PKCδ-mediated pathway which renders the COX-2 expression unresponsive to PKCδ siRNA or rottlerin.

### The action of PKCδ is signaled via ERK1/2

**Figure 8:**
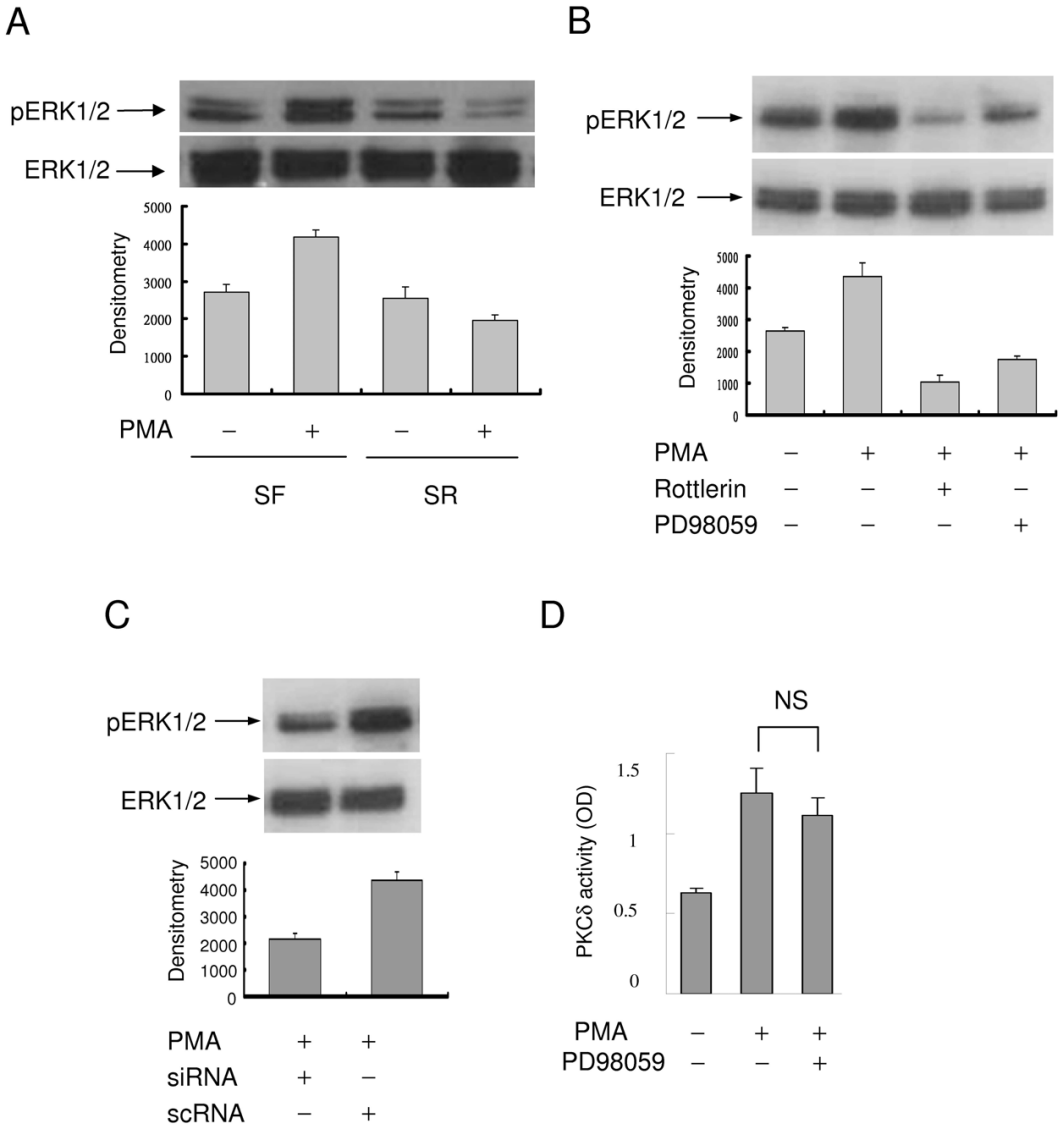
ERK1/2 activation in SF- vs. SR-HsFb. (**A**) Phosphorylated ERK1/2 (pERK1/2) and total ERK1/2 were analyzed by Western blotting. (**B**) SF-HsFb were treated with rottlerin or PD98059 followed by PMA. pERK1/2 and ERK1/2 were analyzed by Western blotting. (**C**) Analysis of pERK1/2 and ERK1/2 in SF-HsFb transfected with PKCδ siRNA or control scRNA. (**D**) SF-HsFb were treated with PD98059 followed by PMA. PKCδ activity was analyzed. Error bars denote mean ± SEM (n = 3). NS denotes statistically insignificant.

Since PMA- and TNFα-induced COX-2 expression was suppressed by the MEK-1/ERK1/2 inhibitor PD in SF- and not in SR-HsFb, we investigated the involvement of ERK1/2 in PKCδ-mediated COX-2 expression in SF-and SR-cells. The basal pERK1/2 was not different between SF- and SR-HsFb (Fig. 8A). PMA increased ERK1/2 phosphorylation in SF-HsFb but not SR-HsFb (Fig. 8A). To determine whether PMA-induced ERK1/2 activation depends on PKCδ activation, we pretreated SF-HsFb with rottlerin or PD and analyzed PMA-induced ERK1/2 phosphorylation. PMA-induced pERK1/2 was blocked not only by PD but also by rottlerin (Fig. 8B), suggesting that ERK1/2 is activated via PKCδ. This was confirmed by PKCδ silencing with siRNA which abrogated PMA-induced ERK activation (Fig. 8C). As PKCδ could also be a downstream signal of ERK1/2, we evaluated the effect of PD on PMA-induced PKCδ activity. PD did not inhibit PKCδ activity (Fig. 8D). Taken together, these results indicate that PKCδ→ERK1/2 signaling pathway plays a pivotal role in governing the differential COX-2 expression in quiescent vs. proliferative fibroblasts. In quiescent cells, COX-2 expression in response to proinflammatory stimuli is mediated via the PKCδ→ERK1/2 pathway and this pathway is blocked in proliferative fibroblasts leading to restrained COX-2 expression.

### Quiescent fibroblasts express a higher level of proinflammatory genes than proliferative fibroblasts

**Figure 9:**
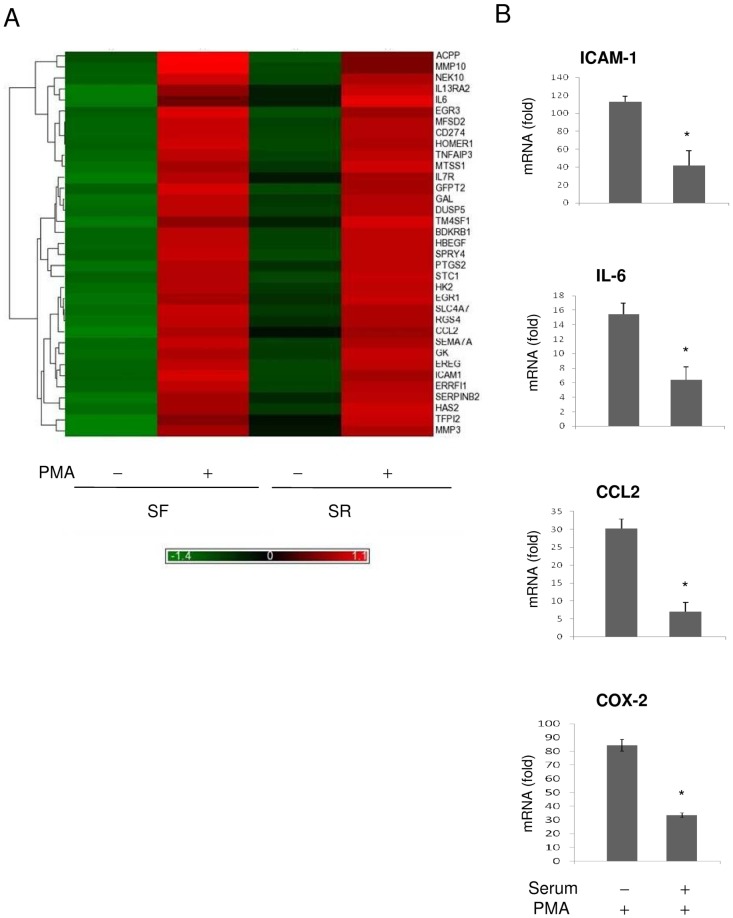
Gene expression profiling in SF- vs. SR-HsFb. (**A**) Heat map shows the genes with differential expression between SF- and SR-HsFb treated with PMA. Gene names are listed at the right margin of the map. (**B**) Validation by quantitative PCR. mRNA levels of several proinflammatory genes in SF- vs. SR-HsFb were measured by qPCR. Error bars are mean ± SEM (n = 3). * *p*<0.05 compared to SF-cells.

We carried out microarray expression assay to identify genes whose expressions are more robust in SF- than in SR-HsFb. RNAs extracted from SF- and SR-HsFb with or without PMA treatment for 4 h were applied to Affimetrix expression microarray. We selected genes whose mRNA levels in PMA-treated SF-HsFb were ≧5-fold higher than that in vehicle-treated SF-HsFb. The mRNA ratio of PMA/vehicle was compared between SF-HsFb and SR-HsFb. 11 genes which included the prototypic gene *PTGS2* (*COX-2*) exhibited a ≧ 2-fold increase of mRNA ratio in SF- over that in SR-HsFb and 24 genes exhibited a 1.5–1.9 fold increase (Table 1). Heat map is shown in Fig. 9A. The list includes many proinflammatory genes. Besides *PTGS2*, it encompasses cytokines and chemokines, i.e. *CCL-2*, *IL-6*, *IL-7R*, *IL-13RA2*, adhesive molecules, i.e. *ICAM-1* and matrix metalloproteinases (MMP), i.e. *MMP-10* and *MMP3*. We performed quantitative PCR to validate the microarray data. The basal mRNA levels of ICAM-1, IL-6 and CCL-2, like that of COX-2 were low in SF- or SR-HsFb and were increased by 15 to >100 fold by PMA stimulation in SF-HsFb (Fig. 9B). PMA-induced mRNA increases were much lower in SR-HsFb, consistent with the microarray data. These results indicate that the expression of a selective group of proinflammatory genes is controlled in a fashion similar to that of COX-2: the expression level is robust in quiescent cells and becomes suppressed in proliferative fibroblasts.

**Table 1:** List of genes with differential expression in quiescent vs. proliferative HsFb.

Gene Symbol	RefSeq	Gene Title	Quiescent/Proliferative
*CCL2*	NM_002982	chemokine (C-C motif) ligand 2	3.6
*SERPINB2*	NM_002575	serpin peptidase inhibitor, clade B	3.3
*IL7R*	NM_002185	interleukin 7 receptor	2.8
*MMP3*	NM_002422	matrix metallopeptidase 3	2.8
*PTGS2*	NM_000963	prostaglandin-endoperoxide synthase 2	2.7
*TFPI2*	NM_006528	tissue factor pathway inhibitor 2	2.5
*IL13RA2*	NM_000640	interleukin 13 receptor, alpha 2	2.4
*RGS4*	NM_001102445	regulator of G-protein signaling 4	2.1
*HAS2*	NM_005328	hyaluronan synthase 2	2.1
*MMP10*	NM_002425	matrix metallopeptidase 10	2.1
*HBEGF*	NM_001945	heparin-binding EGF-like growth factor	2.0
*GAL*	NM_015973	galanin prepropeptide	1.9
*SEMA7A*	NM_003612	semaphorin 7A, GPI membrane anchor	1.9
*ICAM1*	NM_000201	intercellular adhesion molecule 1	1.8
*TM4SF1*	NM_014220	transmembrane 4 L six family member 1	1.8
*ERRFI1*	NM_018948	ERBB receptor feedback inhibitor 1	1.7
*HK2*	NM_000189	hexokinase 2	1.7
*GFPT2*	NM_005110	glutamine-fructose-6-phosphate transaminase 2	1.7
*MFSD2*	NM_001136493	major facilitator superfamily domain containing 2	1.7
*SLC4A7*	NM_003615	solute carrier family 4, sodium bicarbonate cotransporter	1.7
*BDKRB1*	NM_000710	bradykinin receptor B1	1.7
*EGR1*	NM_001964	early growth response 1	1.7
*MTSS1*	NM_014751	metastasis suppressor 1	1.7
*EREG*	NM_001432	epiregulin	1.7
*CD274*	NM_014143	CD274 molecule	1.7
*DUSP5*	NM_004419	dual specificity phosphatase 5	1.7
*SPRY4*	NM_030964	sprouty homolog 4	1.7
*ACPP*	NM_001099	acid phosphatase, prostate	1.6
*STC1*	NM_003155	stanniocalcin 1	1.6
*EGR3*	NM_004430	early growth response 3	1.5
*HOMER1*	NM_004272	homer homolog 1	1.5
*IL6*	NM_000600	interleukin 6 (interferon, beta 2)	1.5
*TNFAIP3*	NM_006290	tumor necrosis factor, alpha-induced protein 3	1.5
*GK*	NM_001128127	glycerol kinase	1.5
*NEK10*	NM_152534	NIMA (never in mitosis gene a)- related kinase 10	1.5

### Quiescent fibroblasts exhibit higher migratory activity than proliferative fibroblasts

**Figure 10:**
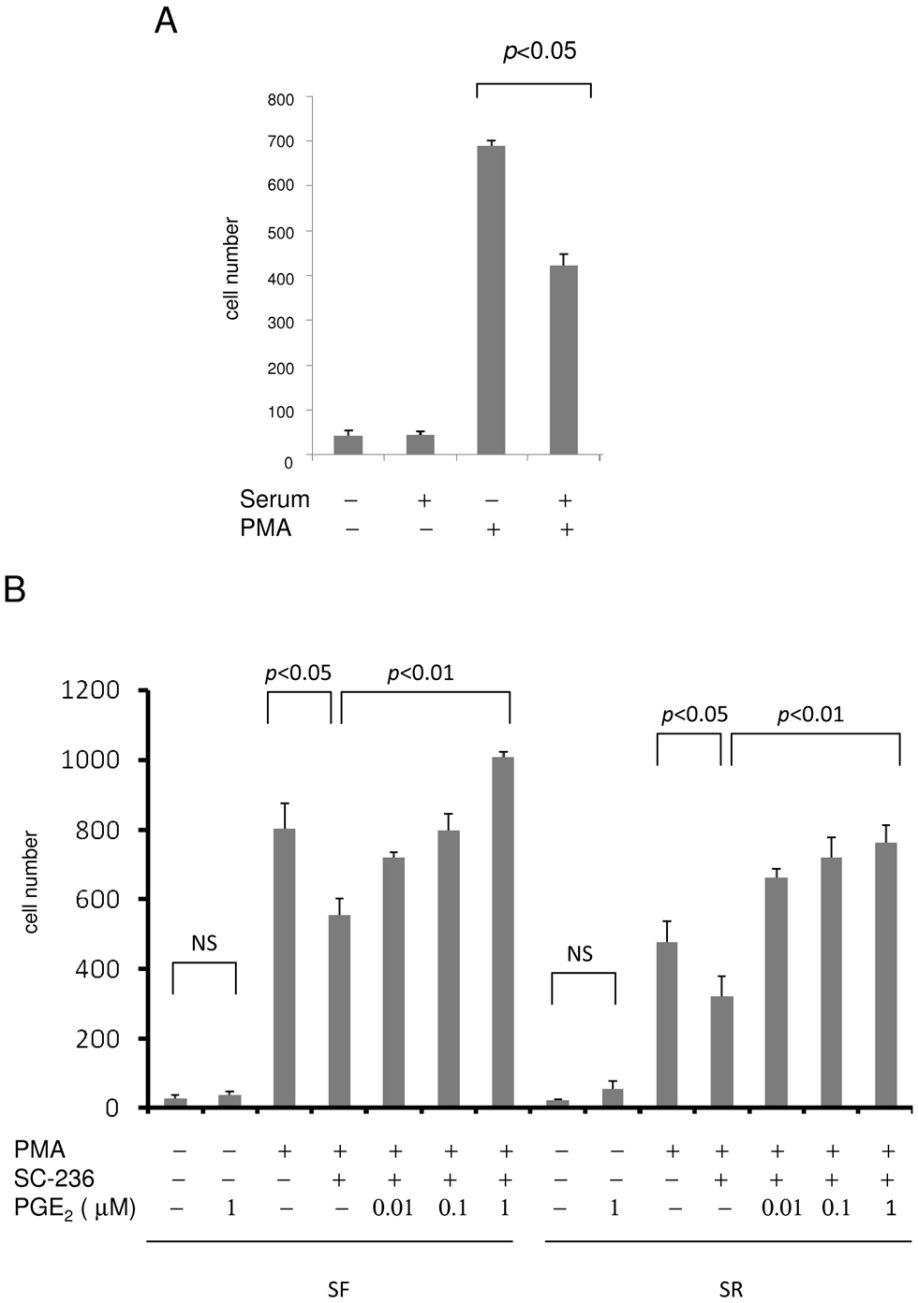
Migration of SF- vs. SR-HsFb. (**A**) SF- or SR-HsFb were treated with PMA for 6 h and cells migrated to the under surface were counted. (**B**) SF-HsFb or SR-HsFb were treated with SC236 (1 uM) for 30 min followed by PMA for 4 h. Cells were applied to the transwell migration assay. For the PGE_2_ rescue experiments, PGE_2_ was added together with SC236 before stimulation with PMA. Error bars refer to mean ± SEM (n = 3).

COX-2 and other proinflammatory genes such as *IL-6* and *MMPs* promote and enhance cell migration [10]. Recent studies provide evidence for migration of fibroblasts from their residence to the injury sites [11], [12]. As SF-HsFb are more responsive to proinflammatory stimuli and express more proinflammatory genes than SR-HsFb, we reasoned that SF-cells could be more migratory than SR-cells. We evaluated fibroblast migration by a transwell assay. Untreated SF- or SR-fibroblasts behaved similarly; only a few cells migrated (Fig. 10A). PMA stimulated HsFb migration; it increased SF-HsFb migration to a higher level than SR-HsFb (Fig. 10A). To determine the extent by which SF- and SR-HsFb migration depends on COX-2, we pretreated cells with SC236, a COX-2 inhibitor and assessed migration. SC236 significantly reduced PMA-induced SF-HsFb migration but only by ∼25% (Fig. 10B). PMA-induced SR-HsFb migration was also reduced by SC236 (Fig. 10B). Reduction of SF- or SR-HsFb migration by SC236 was rescued by PGE_2_ in a concentration dependent manner (Fig. 10B). On the other hand, PGE_2_ had a minimal effect on the basal cell migration in SF- or SR-HsFb (Fig. 10B). These results indicate that SF-HsFb have a higher migration rate than SR-HsFbs when stimulated by PMA. Higher migration response in SF-HsFb is attributed to collective actions of proinflammatory mediators among which COX-2 contribute to migration via PGE_2_ by about 25%.

### Proliferative fibroblasts are less susceptible to PMA-induced DNA oxidation than quiescent fibroblasts

**Figure 11:**
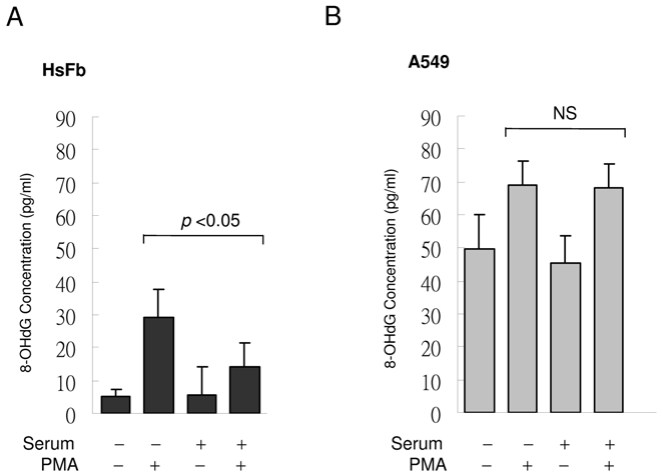
8-OHdG levels in SF- vs. SR-HsFb and A549 cells. (**A**) SF- or SR-HsFb were treated with PMA for 4 h and 8-OHdG was measured. (**B**) SF- and SR-A549 cells were treated similarly to (**A**). Each bar denotes mean ± SEM (n = 3). NS denote statistically non-significant.

It is generally thought that proliferative cells are more risky for DNA oxidation and mutation due to open chromatin structure [13]. As inflammation is a major contributor to DNA oxidation [14], we measured 8-hydroxydeoxyguanosine (8-OHdG), a commonly used surrogate marker of DNA oxidation [15], in SF- vs. SR-HsFb. Basal 8-OHdG was low and not different between resting SF- vs. SR-HsFb (Fig. 11A). PMA increased 8-OHdG in SF-HsFb by ∼6-fold while it increased 8-OHdG by only <4 fold in SR-HsFb (Fig. 11A). For comparison, we analyzed 8-OHdG in A549 lung cancer cells. The basal 8-OHdG level in SF- or SR-A549 cells was at least 10-fold higher than that in SF- or SR-HsFb (Fig. 11B vs. 11A). PMA increased 8-OHdG in SF-A549 by ∼1.4 fold over the basal level and this increase was not significantly different from that in SR-A549 cells (Fig. 11B). These results suggest that the restrained response of proliferative fibroblasts to inflammatory stimuli may protect the cells from DNA oxidation despite its accessibility in the open chromatin structure.

## Discussion

A novel aspect of this study is the demonstration that serum-starved quiescent fibroblasts are highly active in mounting inflammatory responses to exogenous proinflammatory stimuli. They have a more robust expression of diverse proinflammatory genes including *COX-2*, cytokines, adhesive molecules and *MMPs* than serum-driven proliferative cells. As several of the proinflammatory mediators such as COX-2, IL-6 and MMPs are known to promote cell migration, we predicted that quiescent fibroblasts would possess a higher migratory activity than proliferative cells. Our data support this. Since migration is a prerequisite for fibroblasts to contribute to cancer growth [16], wound healing [17], blood vessel remodeling and vascular inflammation [18], our results indicate that quiescent fibroblasts are active in promoting diverse pathophysiological processes by mounting inflammatory responses through robust expression of proinflammatory genes. This new paradigm of inflammation has important physiological implications as more than 50% of cells in the body are at the quiescent state [4]. Diverse quiescent cells including fibroblasts, epithelial cells, endothelial cells, blood cells, and smooth muscle cells may be considered to be at the forefront in defending against insults created by microorganisms, environmental toxins and endogenous immune reactions.

Fibroblasts have been widely used as a cell model to study cell quiescence and cell cycle-dependent cellular changes [3], [4], [8]. They can be rendered quiescent by a number of cell culture manipulations such as serum or growth factor deprivation, contact inhibition or disruption of cell adherence [3]. Using elegant genomic and metabolomic approaches, Coller and associates have characterized quiescent fibroblasts as possessing dynamic gene expressions in an arrest signal-dependent and time-dependent manner [3]. Gene expression profiling reveals a “quiescence program” which contains genes regulating cell division and controlling cell differentiation to ensure non-division and reversibility of the cell cycle arrest [3]. Gene expression profiling further shows that quiescent state of fibroblasts induced by different arrest signals (contact inhibition, serum starvation or non-adhesion) is time-dependent. An overnight cell arrest by one of the quiescent signals results in induction of genes in a signal-dependent manner with a small set of genes regulated irrespective of the arrest signals. This set of commonly regulated genes is expanded when cell arrest by any the arrest signals is prolonged to 4 days. Based on the progression of gene expression profiles, overnight arrest is considered to be “quiescence initiation” and 4-day (and longer) arrest is considered to be at a stage of “quiescence maintenance” [3]. It was unclear whether HsFb at different quiescence states exhibit differential response to exogenous stimuli. To gain insight into this, we analyzed PMA-induced COX-2 expression in HsFb cultured in SF-medium for 24, 48 and 96 h followed by serum replenishing for 24 h. Our results support the notion that serum-starved HsFbs at different quiescence states are reversible of cell cycle arrest and their COX-2 expression in response to PMA stimulation declines to a comparable extent after serum stimulation for 24 h. We also carried out experiments using serum-starved “contact-inhibited” HsFb which will not be driven into S-phase of cell cycle by serum re-stimulation. PMA-induced COX-2 in the contact-inhibited SR-HsFb was as robust as that in SF-HsFb. These findings indicate that once cells are at the quiescent state, COX-2 gene expression is highly responsive to proinflammatory stimulation. After cells have entered the S phase of cell cycle and are actively proliferating, their COX-2 transcriptional activation is restrained through interference of the PKCδ-ERK signaling.

Recent studies have shown that quiescent cells exhibit high metabolic activity with a high rate of glycolysis, pentose phosphate pathway, tricyclic acid flux and NADPH generation [4]. High glucose metabolic rate and NADPH generation may be utilized to carry out cell type-specific functions. In the case of fibroblasts, it was reported that quiescent fibroblasts are robust in producing extracellular matrix to strengthen tissue integrity [4] or antiangiogenic factors such as pigment epithelium-dependent factor (PEDF) for control of excessive new blood vessel formation [19]. Findings from our study suggest that quiescent fibroblasts may direct some of metabolic activity toward response to exogenous stimuli and production of an array of proinflammatory mediators to defend against the invading microorganisms and toxic insults. Furthermore, proinflammatory cytokines and growth factors are actively involved in wound repair and vascular remodeling. When quiescent fibroblasts are driven to proliferation by serum, they change the genetic profile and express pro-angiogenic genes as well as genes that are involved in wound healing [20]. Together with our data, the findings provide a more clear picture about the involvement of fibroblasts in skin wound healing. The quiescent cells are stimulated by the injury (wound) signals to assume an inflammatory and migratory phenotype. At the site of injury, they proliferate and assume a replicating, synthetic phenotype. Through their phenotypic switch, they play a dynamic role in wound healing [17]. A similar quiescent to proliferative phenotypic change occurs in the damaged vascular wall [12]. Through the dynamic phenotypic adaptation, adventitial fibroblasts in blood vessels are increasingly recognized as an important player in blood vessel wall inflammation, remodeling, and vascular repair [18].

Our findings indicate that COX-2 expression in response to proinflammatory stimulation is linked to cell cycle progression. COX-2 expression starts to decline once cells enter into the S-phase of cell cycle. Thus, COX-2 gene expressions is controlled by the cell cycle and cell proliferation program. Expressions of a selective group of proinflammatory genes listed in Table 1 are likely to be controlled by factors involved in cell cycle progression and cell proliferation. Our findings further show that PMA- and TNFα-induced COX-2 expression in quiescent fibroblasts is signaled via the PKCδ→ERK1/2 pathway, which becomes inactive in proliferative fibroblasts. Taken together, these results suggest that certain factor(s) connected to cell cycle progression and cell proliferation suppresses proinflammatory gene expression by inhibiting PKCδ-mediated ERK1/2 activation. It is unclear which factor(s) in proliferative fibroblasts inhibits PKCδ/ERK1/2 and thereby COX-2 expression. We have previously identified a small-molecule factor (designated cytoguardins) in the conditioned medium of proliferative fibroblasts which suppresses proinflammatory mediator-induced COX-2 expression [21]. We suspect that cytoguardins may be generated by proliferative cells to suppress PKCδ/ERK1/2 and COX-2 expression. Work is in progress to test this hypothesis.

Cell proliferation-coordinated reduction of COX-2 and proinflammatory genes expression has important implications in protection against DNA damage. It represents an effective design to reduce the risk of proliferative cell DNA oxidation and gene mutation. COX-2 as well as proinflammatory mediators are reported to induce DNA oxidation through generation of reactive oxygen species [22]. COX-2 inhibitors were reported to attenuate DNA oxidation induced by inflammatory and cytotoxic chemicals [23]. In this study, our results show that corresponding to suppression of COX-2 and other proinflammatory genes in proliferative fibroblasts, PMA-induced 8-OHdG is significantly lower in proliferative fibroblasts compared to quiescent cells. As DNA of proliferative cells is exposed due to open chromatin structure, it is vulnerable to oxidation by reactive oxygen species. An intrinsic cell cycle-dependent control of proinflammatory gene expression represents an important design to reduce DNA damage and gene mutation during DNA replication and cell division.

## Materials and Methods

### Cell culture

Human Hs68 foreskin fibroblasts (HsFb) were obtained from American Type Culture Collection (ATCC), and were cultured as previously described [7]. WI38 cells were obtained from ATCC. A549, MCF7 and MCF10A were obtained from Bioresource Cell Collection and Research Center, Taiwan. They were cultured in DMEM supplemented with 10% fetal bovine serum (FBS) and 1∶100 dilution of antibiotic and antimycotic solution (Invitrogen) at 37°C in a 5% CO_2_ incubator. Cells up to 10 passages were used. To prepare serum-free (SF) cells, cells at 80–90% confluence were washed and incubated in medium without fetal bovine serum (FBS) for 24 h. The serum-replenished (SR) cells were prepared by washing the serum-deprived cells and incubating them in medium containing 2.5% FBS. SF- or SR-HsFb were treated with PMA (100 nM), IL-1β (10 ng/ml), TNFα (10 ng/ml), platelet-derived growth factor BB (PDGF-BB, 10 ng/ml), or fibroblast growth factor 2 (FGF2, 10 ng/ml) at 37°C for 4 h. For treatment with pharmacological inhibitors, cells were pretreated with rottlerin (20 µM), PD98059 (50 µM), or Ly294002 (20 µM) for 30 min prior to addition of PMA or cytokines.

### Western blot analysis

Western blotting was performed as previously described [24]. Western blots were probed with affinity purified rabbit polyclonal IgG against COX-2, cyclin D1, cyclin A, PKCδ or ERK1/2 (Cell Signaling Technology) at 1 µg/ml each. The protein bands were detected by enhanced chemiluminescence (Amersham Pharmacia Biotech) and analyzed by densitometry.

### Assay of PKCδ activity

The immune complex kinase assay was performed using a kit according to manufacturer's instruction (Cell Signaling Technology). In brief, cells were lysed and PKCδ proteins were isolated by immunoprecipitation (IP). PKC activity of the IP-isolated PKCδ was assayed in a 50 µl reaction mixture containing 1.5 µM substrate peptide (CREB biotinylated peptide). The phosphorylated peptide was detected using a sandwich ELISA technique according to manufacturer's instruction.

### PKCδ siRNA transfection

siRNA of PKCδ (5′-CCAUGAGUUUAUCGCCACCTT-3′) and control RNA (scRNA) plasmids were purchased from Santa Cruz. The PKCδ siRNA sequence corresponds to bp715-733 of the coding region of human *PKCδ* (NM-212539.1). HsFb cultured in 6-well plates were transfected with PKCδ siRNA or control plasmids by using lipofectamine 2000 (Invitrogen) as previously described [25]. In brief, siRNA or scRNA plasmids (1 µg DNA in 10 µl) and transfection reagent (5 µl) were incubated in 200 µl serum-free medium for 20 min at room temperature, and the mixture was added dropwise to each well and incubated for 8 h.

### Analysis of COX-2 promoter activity

The promoter activity was determined by transient expression of the COX-2 promoter construct in HsFb by a previously described method [26]. In brief, 4 µg of promoter vector was mixed with 10 µl of lipofectamine 2000 (Invitrogen, Carlsbad, CA). The mixture was slowly added to cells in a 6-well plate and incubated for 24 h. After treatment, cells were lysed and luciferase activity was measured using an assay kit from Promega (Madison, WI) in a luminometer (TD 20/20).

### Chromatin immunoprecipitation (ChIP) assay

The assay was done as previously described [27] with minor modifications. In brief, 1% formaldehyde was added to cells and incubated for 10 min at 37°C. Cells were washed twice in phosphate-buffered saline, scraped, and lysed in lysis buffer (1% SDS, 10 mM Tris-HCl, pH 8.0 with 1 mM phenylmethylsulfonyl fluoride, pepstatin A, and aprotinin) for 10 min at 4°C. The lysate was sonicated, and the debris was removed by centrifugation. One-third of the lysate was used as DNA input control. The remaining two-thirds of the lysate was diluted 10-fold with a dilution buffer (0.01% SDS, 1% Triton X-100, 1 mM EDTA, 10 mM Tris-HCl, pH 8.0, and 150 mM NaCl) followed by incubation with antibodies against p65 and p50, or a non-immune rabbit IgG (Santa Cruz) overnight at 4°C. Immunoprecipitated complexes were collected by using protein A/G plus agarose beads. The precipitates were extensively washed and incubated in an elution buffer (1% SDS and 0.1 M NaHCO_3_) at room temperature for 20 min. Cross-linking of protein-DNA complexes was reversed at 90°C for 10 min, followed by treatment with 100 µg/ml proteinase K for 2 h at 62°C. DNA was extracted three times with phenol/chloroform and precipitated with ethanol. The pellets were resuspended in TE buffer and subjected to PCR amplification using specific COX-2 promoter primers: 5′ primer, ^−709^CTGTTGAAAGCAACTTAGCT^−690^, and 3′ primer ^−32^ AGACTGAAAACCAAGCCCAT ^−51^. The resulting 678 bp product was separated by agarose gel electrophoresis.

### Streptavidin-agarose pulldown (SAP) assay

Binding of transactivators to COX-2 promoter was analyzed by SAP assay as previously described [28], [29]. A biotin-labeled double-stranded probe corresponding to COX-2 promoter sequence −30 to −508 was synthesized by Integrated DNA Technologies (Coralville, IA). A nonrelevant biotinylated sequence 5′-AGAGTGGTCACTACCCCCTCTG-3′ and a −30 to −508 probe in which both κB sites were mutated as previously described [29] were included as a control. The binding assay was performed by mixing 400 µg of nuclear extract proteins, 4 µg of the biotinylated DNA probe and 40 µl of streptavidin-conjugated agarose beads. The mixture was incubated at room temperature for 1 h with shaking, and centrifuged to pull down the DNA-protein complex. DNA-bound p65 and p50 were dissociated and analyzed by Western blotting using specific antibodies.

### Quantitation of mRNA

The mRNA level of COX-2, IL-6, CCL-2 and ICAM-1 were measured by real time quantitative PCR (qPCR). RNA was extracted from HsFb using an RNA extraction kit (Qiagen Biotech). Quality of the extracted RNA was verified at A260/A280 (ratio>1.8) in a spectrometer (ND-1000 Spectrometer, Nanodrop Technologies). cDNA was synthesized from RNA using reverse transcriptase (Invitrogen). For PCR amplification, cDNA and primers were added to SYBR Green PCR Master mix which contains Tag DNA polymerase, dNTPs and SYBR Green 1 dye (Applied Biosystems). The sequence of primers for each gene was obtained by input of the cDNA sequence into Primer 3 database (http://frodo.wi.mit.edu/) and candidate sequences were analyzed in the NCBI Primer-Blast. Primers with the best match were selected for use in amplification. The primer sequences are as follows. COX-2: forward primer (F), 5′-CTGCTCAACACCGGAATTTT-3′ and reverse primer (R), 5′-GAGAAGGCTTCCCAGCTTTT-3′; ICAM-1: F, 5′-GGCTGGAGCTGTTTGAGAAC-3′, and R, 5′-ACTGTGGGGTTCAACCTCTG-3′; CCL-2: F, 5′-CCCCAGTCACCTGCTGTTAT-3′, and R, 5′-TGGAATCCTGAACCCACTTC-3′; interleukin-6 (IL-6): F: 5′-AAAGAGGCACTGGCAGAAAA-3′, and R: 5′-CAGGGGTGGTTATTGCATCT-3′; and reference 18S rRNA: F, 5′-TTGACGGAAGGGCACCACC-3′, and R: 5′-GTCTCGTTCGTTATCGGAATT-3′. Specificity of the amplicons was confirmed by correct size of the band on the electrophoretic gel and correct sequence of the band. Relative quantity of mRNA was determined by the ΔΔCT method as previously described [30] using 18S ribosomal RNA as the internal reference. PMA-induced mRNA levels of COX-2, ICAM-1, CCL-2 or IL-6 in SF- or SR-HsFb were normalized to the basal levels without PMA stimulation. The results were expressed as fold change of PMA-treated vs. untreated cells. Statistical significance was analyzed by paired Student t test using Microsoft Excel software.

### Gene expression profiling by microarray

Amplified and biotinylated sense-strand DNA targets were generated from total RNA using Genechip Whole Transcript (WT) Sense Target Labeling assay system (Affimetrix) according to the user manual. In brief, RNA (100 ng) was reversely transcribed to cDNA and amplified using WT cDNA synthesis and amplification kit. cRNA was degraded with RNase H. Sense strain DNA was cleaned up and fragmented to 40–70 nucleotides by uracil-DNA glycosylase and AP endonuclease I. The fragmented DNA was biotin-labeled using DNA labeling reagent and terminal deoxytransferase (Genechip WT Terminal Labeling kit, Affymetrix Inc.). Biotinylated DNA was hybridized to Affymetrix GeneChip Human Gene 1.0 ST array containing 28,869 human genes (Affymetrix). The array was scanned in Scanner 3000 (Affymetrix). The data were imported to Partek Genome Suite v6.5 (Partek Inc.) and analyzed using Robust Multi-array average. Data from PMA-treated SF-HsFb and SR-HsFb were normalized to vehicle-treated SF-HsFb and SR-HsFb, respectively.

### Cell migration by transwell assay

The migration assay was performed in a 24-well plate in which a 6.5 mm insert (8 µm pore membrane) was placed in each well. SF-HsFb or SR-HsFb treated with or without PMA for 4 h were isolated and seeded on the insert (1×10^4^ cells per well) and incubated in DMEM medium containing 10% FBS at 37°C in a 5% CO_2_ incubator for 6 h. Cells migrated to the lower surface of the insert were stained and counted under microscopy. The results were expressed as cell counts per 10 fields.

### Assay of 8-OHdG

DNA was extracted from cellular pellets using Genomic DNA purification kit (Promega) and incubated with nuclease P1 (Sigma-Aldrich, St. Louis, MO, USA) for 2 h at 37°C in acetate buffer, pH 5.3. Nucleotides generated by hydrolysis were treated with alkaline phosphatase (Sigma-Aldrich) in Tris-HCl buffer, pH 8.0 at 37°C for 1 h. The samples were boiled for 10 min and placed on ice. 8-OHdG was assayed using an EIA kit (Cayman) according to the manufacturer's instruction. In brief, plates pre-coated with goat anti-mouse IgG were blocked with a protein solution provided with the kit. 8-OHdG standards or samples were added to the pre-coated plate, and incubated with 8-OHdG-linked acetylcholinesterase, 8-OHdG monoclonal antibody and sample at 4°C for 18 h. After washing, Ellman's reagent was added to each well and the absorbance at 412 nm was read in a spectrophotometer. The 8-OHdG content was determined from a calibration curve.

### Statistical analysis

The statistical significance of differences between the experimental and control groups was assessed by paired Student t test using a Microsoft Excel program. The statistical significance of multigroup differences was analyzed by ANOVA. A *p* value less than 0.05 was considered to be statistically significant.
